# The whitefly puparia from Cretaceous amber reveal early subfamily diversification

**DOI:** 10.7717/peerj.21463

**Published:** 2026-07-08

**Authors:** Alejandro Caballero, Jowita Drohojowska, Gregory Evans, Jacek Szwedo, Chenyang Cai, Dolev Fabrikant, Diying Huang, Yanzhe Fu

**Affiliations:** 1 Ludwig-Maximilians-Universität Muünchen, Biocenter, Planegg-Martinsried, Germany; 2 Bavarian State Collection of Zoology (SNSB–ZSM), Section Hemiptera, München, Germany; 3 Institute of Biology, Biotechnology and Environmental Protection, Faculty of Natural Sciences, University of Silesia, Katowice, Poland; 4 USDA/APHIS/PPQ c/o Systematic Entomology Laboratory, USDA, Beltsville, United States of America; 5 Laboratory of Evolutionary Entomology and Museum of Amber Inclusions, Department of Invertebrate Zoology and Parasitology, Faculty of Biology, University of Gdańsk, Gdańsk, Poland; 6 State Key Laboratory of Palaeobiology and Stratigraphy, Nanjing Institute of Geology and Palaeontology, Nanjing, China

**Keywords:** Cretaceous, Fossil, New species, Aleyrodidae, Morphology, Puparia

## Abstract

Morphological information on fossil whiteflies has been largely limited to adult specimens. Described puparia fossils provide little morphological information due to the fossilisation process on substrates and the main characters to place them in a subfamily are missing. Here, we describe and illustrate three extinct species: *Palaeoaleuroclava grandirostrata* gen. et sp. nov., *Cretacerifera mranmaense* gen. et sp. nov. (subfamily Aleyrodinae), and *Cenomaleurodicus multiporu* gen. et sp. nov. (subfamily Aleurodicinae), based on puparia preserved in mid-Cretaceous Myanmar amber (ca. 100 Mya). These fossils represent the first known whitefly puparia with visible dorsal and ventral surfaces and wax traces, the first record of a specimen of the subfamily Aleurodicinae, and the only known occurrence from the Mesozoic era. The morphological information available in the specimens’ fossils allows discussion of the evolution of the subfamilies Aleyrodinae and Aleurodicinae, based on the presence of claws and compound pores, and the proportions of the lingula. Comparative analysis reveals that several key morphological features observed in extant genera evolved by the mid-Cretaceous, providing new insights into the early evolution and diversification of whiteflies during the late Mesozoic.

## Introduction

Hemiptera is the largest non-holometabolous insect order, characterised by extensive morphological and ecological diversity, with more than 100,000 extant species described (Henry, 2017; Bartlett Charles et al., 2018; Song et al., 2024). Sternorrhyncha, considered the sister group to the remaining hemipterans based on morphological and molecular evidence, contains the major extant lineages Psylloidea (psyllids and relatives), Aleyrodomorpha (whiteflies), Aphidomorpha (aphids, phyloxerans, adelgids and relatives), and Coccidomorpha (scale insects) (Cryan & Urban, 2012; Li et al., 2017; Johnson et al., 2018). Almost all members of Sternorrhyncha are entirely phytophagous, feeding on plant sap typically extracted from the phloem or, rarely, mesophyll, and many exhibit a strong association with specific host plants (Grimaldi & Engel, 2005; Hardy, 2018).

Whiteflies encompass a group of small, soft-bodied, and plant-sap-sucking insects known for their economic importance as pests. Compared to aphids and scale insects, modern whiteflies appear to be a less species-rich group, with about 1,700 known species (Martin & Mound, 2007; Drohojowska, Śladowska & Szwedo, 2024). Whiteflies typically have a layer of wax and undergo four larval stages in their life cycle, with the final (fourth) larval instar commonly known as the ‘puparium’ (Quaintance & Baker, 1913; Gill, 1990). Currently, Aleyrodomorpha is composed of the sole family Aleyrodidae Westwood, 1840, based on the presence of a vasiform orifice in all development stages (Martin, 2004), and supported by molecular analysis (Zhao et al., 2025). Aleyrodidae is divided into four subfamilies: one extinct, Bernaeinae Shcherbakov, 2000, and three extant: Aleyrodinae Westwood, 1840, Aleurodicinae Quaintance & Baker, 1913, and Udamoselinae Enderlein, 1909 (Quaintance & Baker, 1913; Quaintance & Baker, 1914). Bernaeinae and Udamoselinae were established based on adult specimens, and there is no information about their pupal stages (Shcherbakov, 2000; Martin, 2007). In contrast, the morphology of the puparia stage of Aleyrodinae and Aleurodicinae has been largely used to build the taxonomy of both groups. At the subfamily level, the diagnostic features to differentiate both subfamilies rely on characters of antennae, legs, vasiform orifice, lingula and dorsal pores (Martin, 1999; Martin, Mifsud & Rapisarda, 2000; Gullan & Martin, 2009).

The fossil record of Aleyrodidae ranges from the Late Jurassic to the Pliocene, and fossil-based contributions to the taxonomy of the family have largely relied on adult specimens, with about 30 species described (Shcherbakov, 2000; Martin, Mifsud & Rapisarda, 2000; Drohojowska & Szwedo, 2011a; Drohojowska & Szwedo, 2011b; Drohojowska & Szwedo, 2013; Drohojowska & Szwedo, 2015; Drohojowska, Perkovsky & Szwedo, 2015; Szwedo & Drohojowska, 2016; Szwedo et al., 2019; Drohojowska et al., 2019; Drohojowska et al., 2020; Drohojowska et al., 2023; Drohojowska et al., 2024; Drohojowska et al., 2025; Chen et al., 2020; Chen et al., 2021; Chen et al., 2022; Drohojowska, Śladowska & Szwedo, 2024; Drohojowska, Zmarzły & Szwedo, 2024; Drohojowska, Franielczyk-Pietyra & Szwedo, 2025). Most of these fossils are preserved in amber, which allows the observation of relevant characters, such as wing venation, that are essential for taxonomic interpretation (Szwedo & Drohojowska, 2016). This contrasts with the reduced number of species described from puparium fossils. As it is detailed below, only three valid fossil species have been described from puparia, all preserved as compression fossils from the Neogene, with visible anatomy restricted to the dorsal surface because the parent rock obstructs the ventral view. The fact that most of the taxonomic characters rely on the ventral surface and dorsal cuticular structures means that the matrix in which a puparium specimen is fossilised is crucial for its analysis.

There are three described species, based on puparia fossils: *Aleurochiton petri* Rietchel, 1983, *Praealeurolobus indicus* Drohojowska et al., 2023, and *Miotetraleurodes novaezelandiae* Drohojowska et al., 2024, all of them placed in the subfamily Aleyrodinae (Rietschel, 1983; Drohojowska et al., 2023; Drohojowska et al., 2024). Aside from the three valid Neogene fossil species of whitefly puparia, several older but taxonomically uncertain records have also been reported. An Early Pliocene sample from the Beaver peat, Ellesmere Island in the Canadian Arctic preserve several specimens of whiteflies that could only be determined to the infraorder level (Matthews & Telka, 1997). A similar case occurs with unidentified puparia of Aleyrodidae from the Upper Eocene (Priabonian) Bembridge Marls Member of the Bouldnor Formation, Isle of Wight, United Kingdom (Jarzembowski & Ross, 1993; Szwedo et al., 2019). Additionally, a slightly older puparium fossil was found on a leaf surface from the Middle Eocene Fossil Lagerstätte Geiseltal in Germany (Weigelt, 1940). This fossil was named ‘*Aleurochiton eozaenicus*’, but Weigelt provided no diagnosis or other features which could allow the recognition of the identity of this fossil; therefore, the name was recognised as a *nomen nudum* (Drohojowska & Szwedo, 2015). Finally, the oldest puparium fossil comes from the Purbeck Limestone Group of southern England, which consists mainly of lagoonal deposits of the Early Cretaceous (Jarzembowski & Coram, 1997; Coram & Jarzembowski, 2021). Nevertheless, the specimens remain attached to the parental rock, which, as in previous cases, has the ventral surface blocked. Consequently, classification could not be refined beyond Aleyrodomorpha, based on the preserved remnants of the vasiform orifices (Jarzembowski & Coram, 1997; Coram & Jarzembowski, 2021).

Another relevant difference between puparium and adult fossils relies in their age. The accepted species described from puparia fossils have been dated between the Miocene and the Pliocene epochs (23 to 3 Mya), while adult fossils can be traced back to the Upper Jurassic (Drohojowska et al., 2019). Mesozoic whiteflies comprise 14 formally described species, most of them distributed throughout Asia (Chen et al., 2022; Drohojowska, Franielczyk-Pietyra & Szwedo, 2025). They are found in sedimentary deposits from the Middle Jurassic Daohugou beds in China and the Upper Jurassic deposits of Mongolia and Kazakhstan, as well as the fossil resins from the Cretaceous amber of Lebanon and Myanmar (Shcherbakov, 2000; Drohojowska & Szwedo, 2011a; Drohojowska et al., 2019; Chen et al., 2020; Chen et al., 2022).

Here, we describe and illustrate three fossil puparia, from Kachin amber, Myanmar, providing a full venter-dorsum description. The newly discovered inclusions represent the first whitefly puparia from the Mesozoic, providing new insights into the fossil record of the ancient Sternorrhyncha lineage. One of these amber-preserved specimens represents the first fossil representative of Aleurodicinae, and its morphology is compared with that of extant species.

## Materials & Methods

### Studied material

The studied amber pieces originated from amber mines near Noije Bum Hill, in the Hukawng Valley, Myitkyina District, Kachin State, Myanmar. Hukawng Valley sites in Kachin State have been mined for centuries (Kania, Wang & Szwedo, 2015; Thu & Zaw, 2017; Jiang, Szwedo & Wang, 2018). This resin was designated as burmite (Helm, 1892; Helm, 1893; Noetling, 1893) and is now commonly referred to as Kachin amber. The amber-bearing deposits manifest as clastic sedimentary layers, characterised by thin limestone beds and abundant coaly and carbonaceous material. Radiometric dating of the amber-bearing horizon revealed an age of 98.79 ± 0.62 Ma (Shi et al., 2012). Additionally, paleontological evidence, including ammonites, foraminiferans, insects, and sporo-pollen assemblages, suggests that the amber is generally dated to the Albian–Cenomanian boundary (Cruickshank & Ko, 2003; Rasnitsyn et al., 2016; Smith & Ross, 2017; Mao et al., 2018; Yu et al., 2019).

To avoid any potential confusion or misunderstanding, it must be clarified that all contributing authors hereby declare that the fossils in question were obtained legally prior to June 2017. Furthermore, the fossils were categorically not involved in any armed conflict or ethnic strife in Myanmar. The specimens NIGP205888 and NIGP205889 are deposited in Nanjing Institute of Geology and Palaeontology, Chinese Academy of Sciences, Nanjing, China. The specimen PED3058 is deposited in the Palaeo-Evo-Devo Research Group Collection of Arthropods, Ludwig-Maximilians-Universität München (LMU Munich), Germany, in full compliance with the International Code of Zoological Nomenclature (ICZN, 1999), the Statement of the International Palaeoentomological Society (Szwedo et al., 2020) and the policies presented by Haug et al. (2020).

### Analytical methods

Bright-field images were taken with a Zeiss Discovery V16 stereo microscope and a Keyence VHX-6000 digital microscope. Focus-stacking software (Helicon Focus 7.0.2) was used to increase the depth of field. Line drawings were drafted in Adobe Illustrator and Adobe Photoshop. Morphological measurements were conducted using ImageJ software (Abramoff, Magalhaes & Ram, 2004; Schneider, Rasband & Eliceiri, 2012). Morphological terminology is based on Martin (1987), Martin (1999) and Martin (2005); the terms ‘glandular seta’ and ‘glandular spine’ are treated indistinctly and we will use the first term.

The electronic version of this article in Portable Document Format (PDF) represents a published work according to the International Commission on Zoological Nomenclature (ICZN). Consequently, the new names contained in the electronic version are effectively published under that Code from the electronic edition alone. This published work, along with the nomenclatural acts it contains, has been registered in ZooBank, the online registration system for the ICZN. The ZooBank LSIDs (Life Science Identifiers) can be resolved and the associated information viewed through any standard web browser by appending the LSID to the prefix http://zoobank.org/. The LSID for this publication is: http://zoobank.org/urn:lsid:zoobank.org:pub:pub:8E9CE1A7-2833-440F-B9E9-BBCE5B83ABC4. The online version of this work is archived and available from the following digital repositories: PeerJ, PubMed Central SCIE and CLOCKSS.

## Results

### Systematic palaeontology

**Table:** 

Order Hemiptera Linnaeus, 1758
Suborder Sternorrhyncha Amyot et Audinet-Serville, 1843
Family Aleyrodidae Westwood, 1840
Subfamily Aleyrodinae Westwood, 1840

**Table:** 

Genus *Palaeoaleuroclava* gen. nov.

Type species. *Palaeoaleuroclava grandirostrata* sp. nov.; here designated.

LSID: urn:lsid:zoobank.org:act:50847E47-A3B7-4D91-9989-5B366ED4DA47

Etymology. The generic name is a combination of the Ancient Greek ‘*π*αλα*ι*ó*ς*’ (*palaiós*) meaning old, or ancient, and the generic name *Aleuroclava* Singh, 1931. Gender: feminine.

Diagnosis. Submarginal area and dorsal disc separated by a visible line on the prosoma; vestigial leg pads without segmentation, cephalothoracic suture strongly sclerotised on the anterior central area of the dorsum, with anterolateral extensions reaching the line that divides the submarginal area and the dorsal disc.

**Table:** 

**Figure 1:**
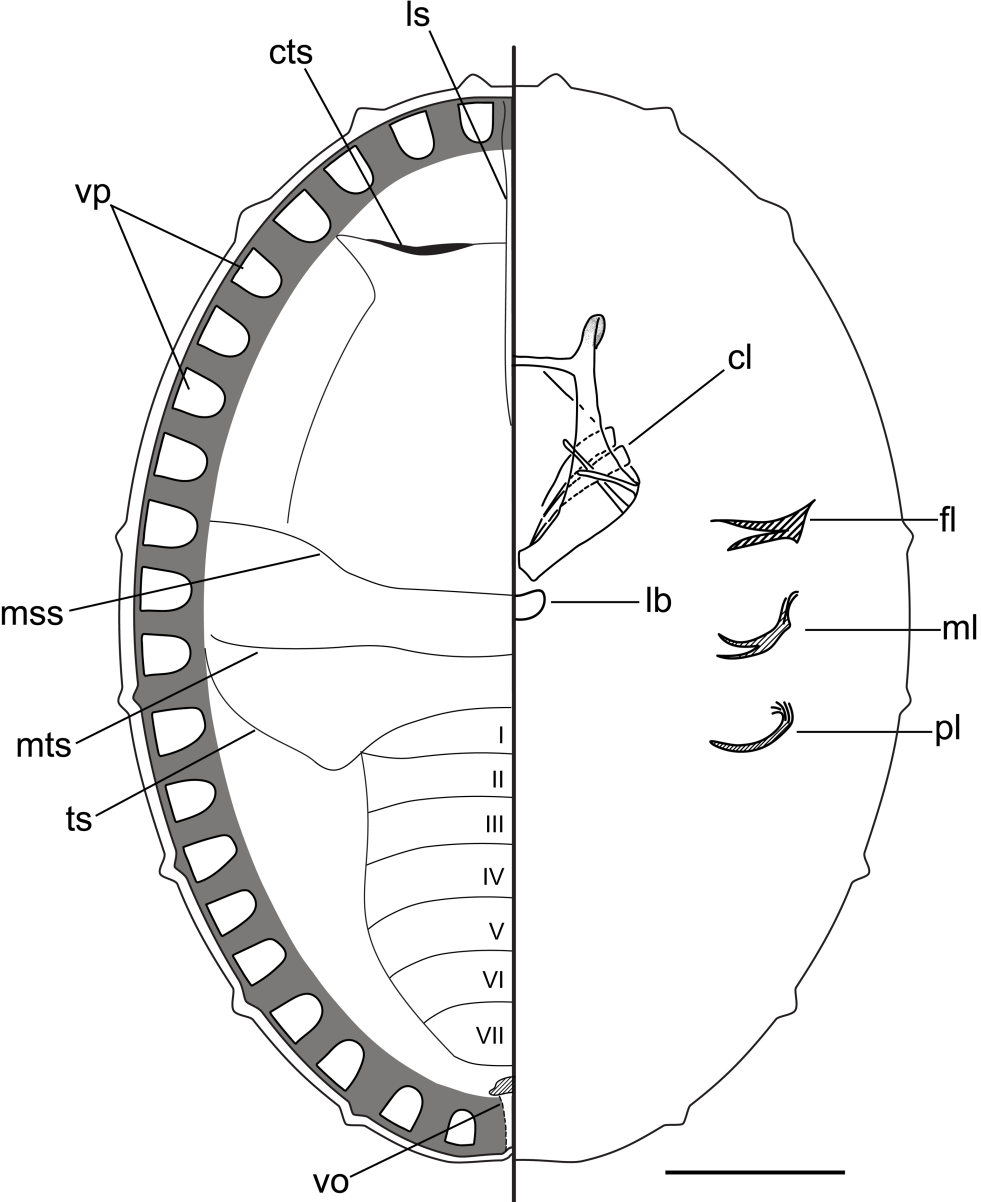
Reconstruction drawing of *Palaeoaleuroclava grandirostrata* gen. et sp. nov., puparium specimen with dorsal view (left side) and ventral view (right side). Abbreviations in alphabetical order: abdominal segments as roman numbering, (cl) clypeolabral shield, (cts) cephalothoracic suture, (fl) vestigial foreleg, (lb) labium, (ls) longitudinal suture, (ml) vestigial midleg, (mss) mesothorax suture, (mts) metathorax suture, (pl) vestigial posterior leg, (ts) transversal suture, (vo) vasiform orifice plus abdominal numeration as roman numerals, (vp) vestigial papillae. Scale bar = 50 µm.

**Figure 2:**
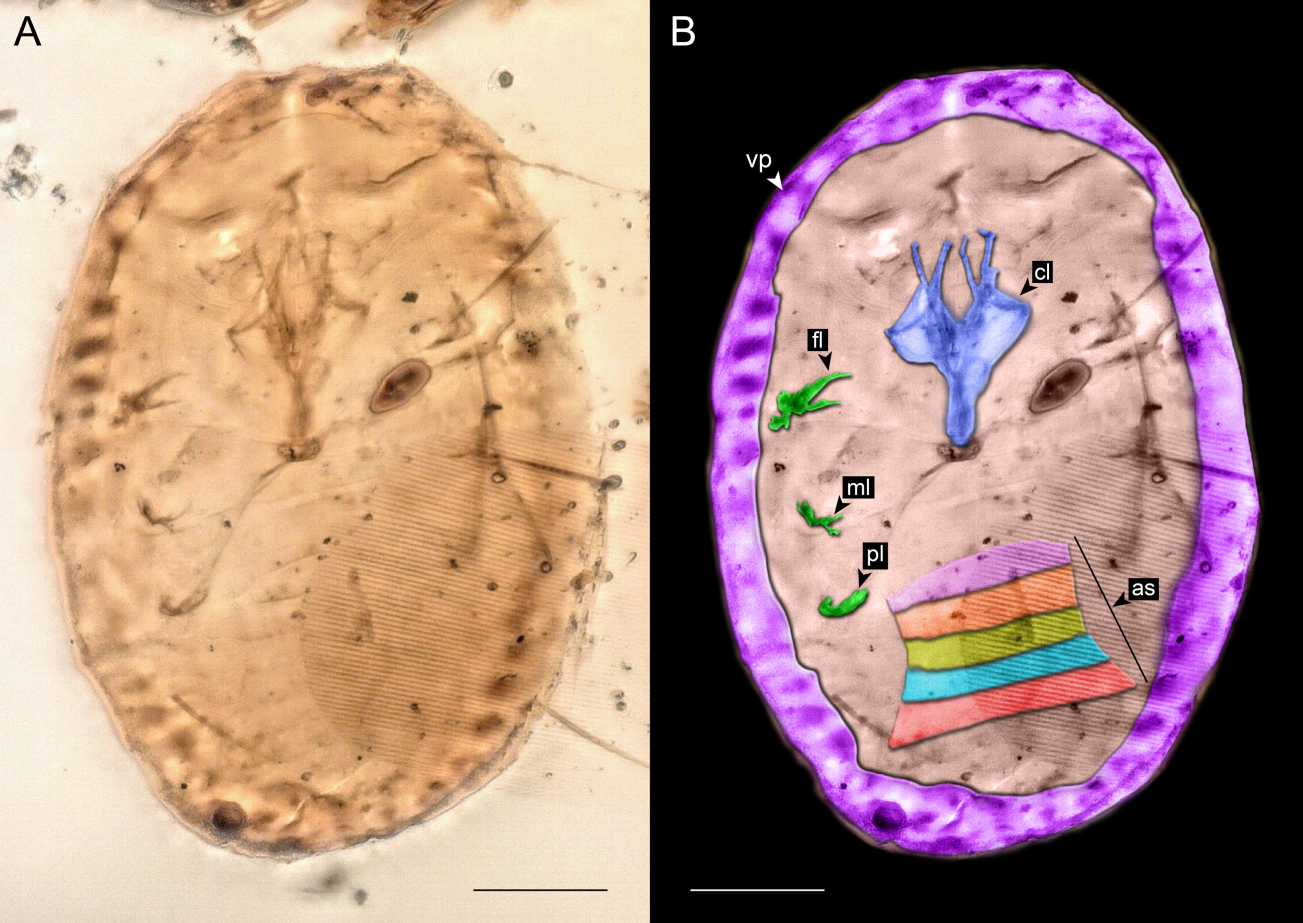
Microphotographs of *Palaeoaleuroclava grandirostrata* gen. et sp. nov. (A) Wide view of body without coloured marks. (B) Microphotograph with colored shadows indicating (as) abdominal segments, (cl) clipeo-labral shield, (fl) foreleg, (lb) labium, (ml) midleg, (pl) posterior leg, (vp) vestigial papillae. Scale bar = 50 µm.

*Palaeoaleuroclava grandirostrata* sp. nov.
Figs. 1 and 2

LSID: urn:lsid:zoobank.org:act:46856C02-90A4-453A-9E49-E19B8B7AB8AB

Etymology. The specific epithet *grandirostrata* is a combination of the Latin adjective ‘grandis’, meaning large, great, or grand, and the noun ‘rōstrum’ (snout, beak), referring to the unusually large size of the mouthparts.

Diagnosis. Large rostrum, covering nearly one-quarter of the length of the body. Antennae absent. Operculum reduced to a small sclerotised spot. Submarginal area with a band of sclerotised patches.

Holotype. PED3058: A puparium from Hukawng Valley, Kachin State, Myanmar; lowermost Cenomanian, Upper Cretaceous; deposited in the Palaeo-Evo-Devo Research Group Collection of Arthropods, Ludwig-Maximilians-Universität München (LMU Munich), Germany.

Description. The whitefly puparium is enclosed in a transparent, yellowish amber piece (collection of the Palaeo-Evo-Devo research group), without a trace of the vegetal host. Additionally, the amber contains a lacewing larva (Neuroptera) as a synbioinclusion.

Puparium (Fig. 1 & Figs. 2A–2B): Body broadly oval with anterior and posterior margins flattened, 294 µm long and 212 µm wide, pale in colour. *Dorsum*–Margin with few peaks, with a narrow transparent band. Submarginal area with around 20 vestigial papillae (Fig. 1, vp; Fig. 2B, vp) on each half, along the surface. Line partially visible on the prosoma separating the submarginal area and the dorsal disc. Longitudinal suture (Fig. 1, ls) from the margin of the head to the level of the anterior section of the clypeolabral shield. Cephalothoracic suture (Fig. 1, cts) extended transversally from the submargin to the lateral areas of the longitudinal suture, 27–29 µm long. Meso- (Fig. 1, mss), meta- (Fig. 1, mts) and transversal sutures (Fig. 1, ts) visible. Length of mesothorax and metathorax along midline of 20 µm and 22 µm, respectively. Abdominal segments with visible sutures separating them (Fig. 1B, as), lengths of segments along midline as follows: I and II 13 µm, III 11 µm, IV 13 µm, V and VI 10 µm, VII 11 µm, VIII 5 µm. Vasiform orifice (Fig. 1, vo) is not clearly defined, with operculum as a dark spot located in the internal border of the submarginal band. Lingula is not differentiable. Caudal furrow barely visible, enclosed in the submarginal band with a notch on the posterior margin, 9 µm long. Compound pores, setae or tubercles absent. *Venter*–slightly concave, antennae not visible, clypeolabral shield (Fig. 1, cl; Fig. 2B, cl) 57 µm long and 55 µm wide, with anterior and posterior arms visible. Labium (Fig. 1, lb) 9 µm long and 17 µm wide. Vestigial legs, no segmentation recognisable, lengths as follows: foreleg (Fig. 1, fl; Fig. 2B, fl) 23 and 25 µm, mid leg (Fig. 1, ml; Fig. 2B, ml) 26 and 18 µm and posterior leg (Fig. 1, pl; Fig. 2B, pl) 99 and 96 µm. *Setae arrangement–* not observed

Remarks. *Palaeoaleuroclava grandirostrata* is placed in Aleyrodinae because it lacks compound pores. The legs and the vasiform orifice are quite reduced, therefore, information of claw and lingula characters are not visible.

The large size of the rostrum in *P. grandirostrata* seems not to be a common feature in extant species. Most of the literature, including the largest cladistic analysis of subfamily Aleyrodinae and the Whitefly Pupa of the World database, does not include this trait as a taxonomic feature (Manzari & Quicke, 2006; Dooley, 2011). However, a proportion comparison based on photographs and illustrations allows an approximate contrast between species. Considering the sum of clypeolabral shield and labium, the rostrum:body proportion of *P. grandirostrata* is about 1:4.5. The closest case is *Palaealeurodicus holmesii* (Maskell, 1986), with a ratio about 1:5 (see Martin, 1999, p. 139), although that species presents a bi-segmented labium. *Trialeurodes eriodictyonis* Russell, 1948 and *T*. *euphorbiae* Russell, 1948 present a large rostrum proportion, about 1:5.6 and 1:5.7, respectively (see *Trialeurodes* section on Dooley, 2011). Another species, *Synaleurodicus hakeae* Solomon, 1935, has a ratio about 1:5.7 (see Martin, 1999 p. 142), although that species belongs to the subfamily Aleurodicinae.

The other relevant character in *P. grandirostrata* is the vestigial papillae in a submarginal band. This feature is common in two main groups of species: a New World group comprising *Trialeurodes* Cockerell, 1902, *Xenaleyrodes* Takahashi, 1936, and *Trialeurolonga* Martin, 2005; and an Oriental-Palaearctic group including *Dialeurolonga* Dozier, 1928 and *Aleuroclava* Singh, 1931 (Martin, 2005; Manzari & Quicke, 2006; Evans, 2008; Pushpa & Sundararaj, 2010). Among these genera, four species possess a similar distribution, abundance and approximate arrangement of vestigial papillae, while lacking papillae in any other area of the dorsum: *Trialeurodes ruborum* (Cockerell, 1896), *T. variabilis* (Quaintance, 1900), *Aleuroclava tianmuensis* Wang & Dubey, 2014, and *A. aucubae* Kuwana, 1911.

Morphologically, *T. ruborum* and *T. variabilis* have the simplest composition of papillae (Russell, 1948), which may resemble the simplicity of the vestigial papillae found in the fossil. Regarding *A. tianmuensis* and *A. aucubae*, *P. grandirostrata* shares several key morphological traits, including the presence of clear areas in the submarginal region resembling tuberculate papillae, vestigial and unsegmented legs, and a modified dorsal cephalothoracic suture with lines extending anterolaterally toward the submargin, plus the geographical distribution (Wang, Dubey & Du, 2014; Gavrilov-Zimin & Borisov, 2020). However, this fossil is distinguished from those species by two unique features: (i) an unusually large rostrum, extending to cover one-quarter of the body length; and (ii) a notably small anal apparatus, where the vasiform orifice is barely visible, the operculum is reduced to a sclerotised spot, and the lingula is indistinct, possibly due to significant degradation of the specimen.

Geographically, *Trialeurodes* comprises about 100 species, with a distribution mainly in the New World; *T. ruborum* and *T. variabilis* are restricted to the tropical region (Russell, 1948; Evans, 2008). In contrast, *Aleuroclava* includes approximately 124 species, with around 100 species documented from the Oriental and Australian regions (Martin & Mound, 2007). In Myanmar specifically, there are three species recorded: *Aleuroclava burmanicus* Singh, 1938, *Aleuroclava nitidus* (Singh, 1932), and *Aleuroclava parvus* (Singh, 1938) (Dubey & David, 2012). However, neighbouring countries India and China report significantly higher diversity, with 69 and 35 species, respectively (Pushpa & Sundararaj, 2010; Wang, Xu & Zhou, 2020). Although *P*. *grandirostrata* shows morphological features of both *Trialeurodes* and *Aleuroclava* species, the geographical aspect might indicate an evolutionary relationship with *Aleuroclava*.

**Table:** 

Genus: *Cretacerifera* gen. nov.

Type species. *Cretacerifera mranmaense* sp. nov. here designated.

LSID: urn:lsid:zoobank.org:act:BA3A9BAD-75DC-4403-B185-84987D2B5C88

Etymology. The generic name is a combination of the Latin noun ‘*crēta*’ –chalk, indicating the geological period, and the Latin noun ‘*cēra*’ –wax and the Latin verb ‘*ferō*’ –to bear, and refers to the wax filaments. Gender: feminine.

Diagnosis. Big vestigial pores (22–38 µm) and a single row of papillae are present on the submargin of the dorsum.

**Table:** 

**Figure 3:**
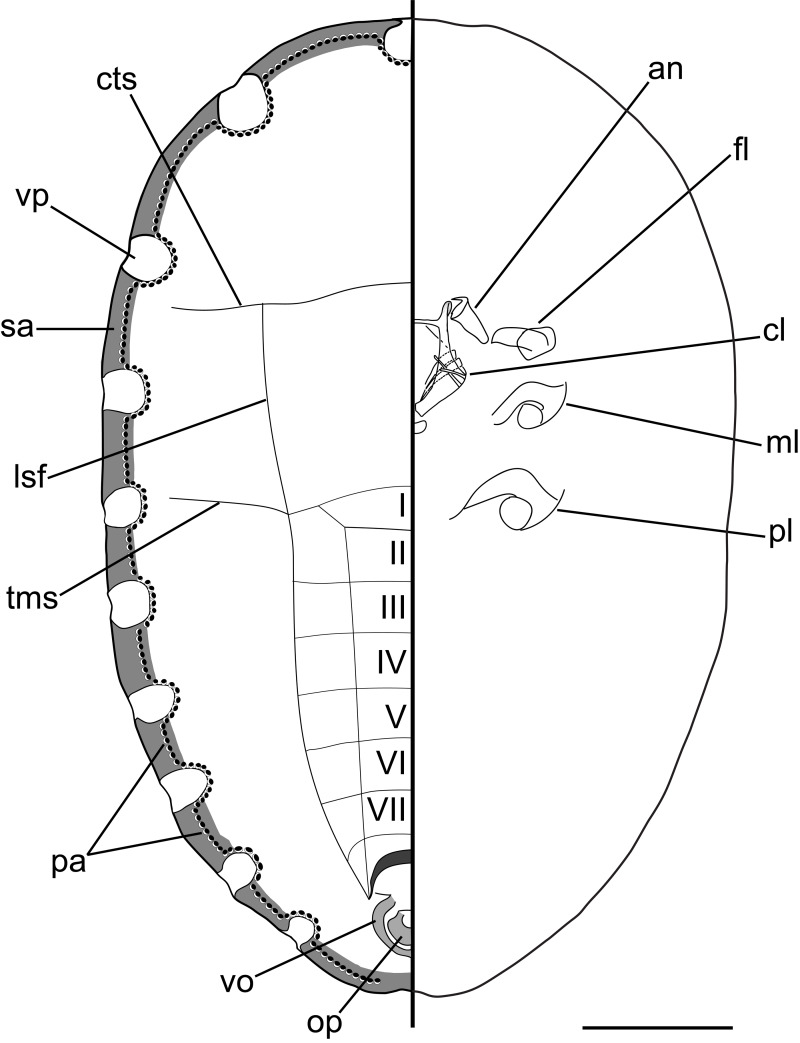
Reconstruction drawing of *Cretacerifera mranmaense* gen. et sp. nov., a puparium specimen with dorsal view (left side) and ventral view (right side). Abbreviations in alphabetical order: abdominal segments as roman numbering, (an) antenna, (cl) clipeolabral shield, (cts) cephalothoracic suture, (fl) vestigial foreleg, (lsf) longitudinal subdorsal furrow, (ml) vestigial midleg, (pa) papillae, (pl) vestigial posterior leg, (op) operculum, (sa) submarginal area, (tms) transversal moulting suture, (vo) vasiform orifice, (vp) vestigial pores. Scale bar = 100 µm.

**Figure 4:**
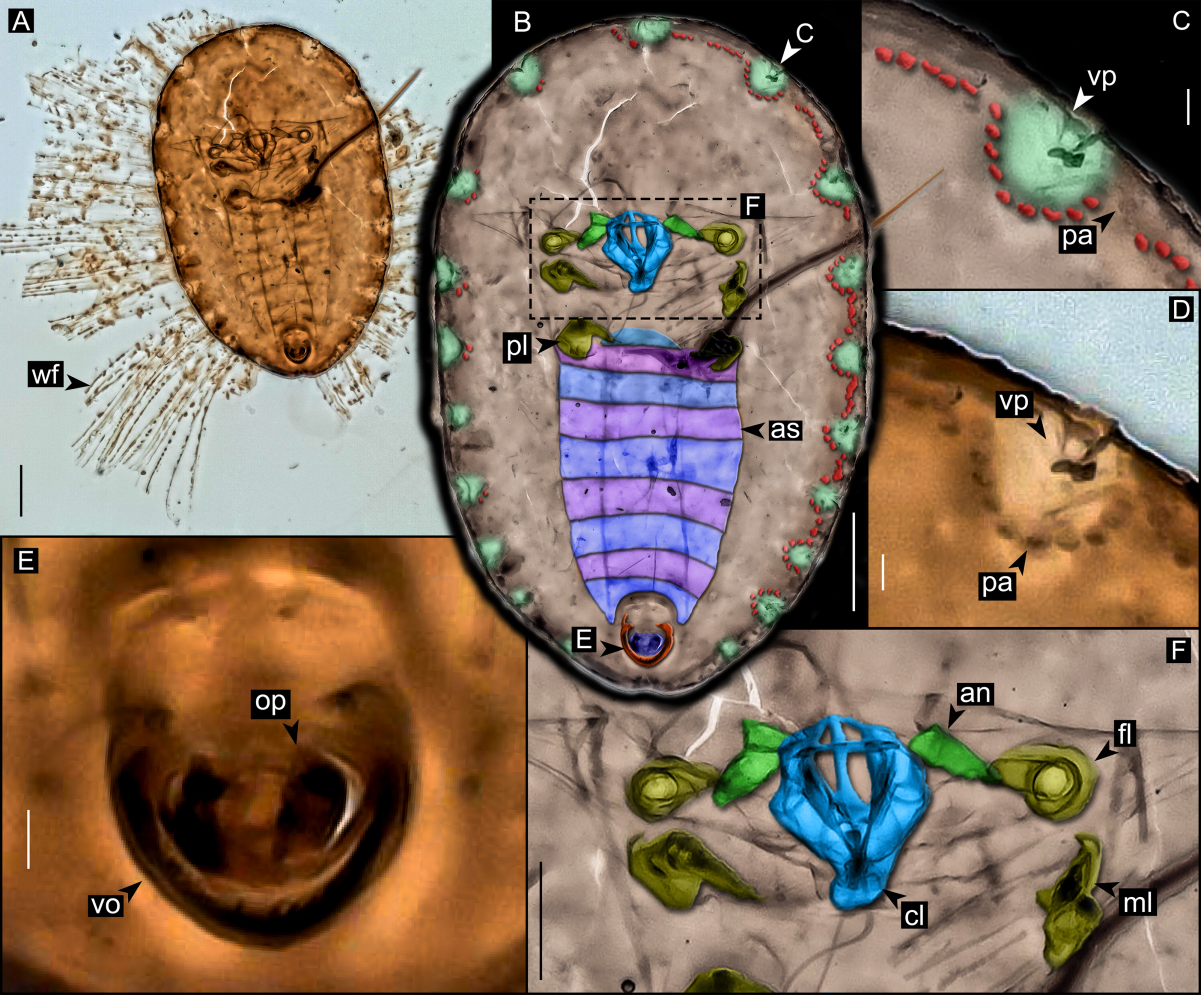
Microphotographs of *Cretacerifera mranmaense* gen. et sp. nov., a puparia specimen. (A) Wide view of body without coloured marks, indicating the (wf) wax filaments. (B) Body with coloured shadows indicating (as) abdominal segments, (cl) clipeo-labral shield, (fl) foreleg, (ml) midleg, (pl) posterior leg, (sa) submarginal area, (vo) vasiform orifice, (vp) vestigial pores. (C) High-contrast picture of full body (left side) and close-up of (pa) papillae. Scale bar: A, B = 100 µm; C–E = 10 µm; F = 50 µm.

*Cretacerifera mranmaense* sp. nov.
Figs. 3 & 4

LSID: urn:lsid:zoobank.org:act:E1FBA0DF-041C-4D0C-856D-9F530325B9C7

Etymology. The specific name is derived from the Burmese ethnonym Mranma and refers to the English name of the country of Myanmar.

Diagnosis. The species is differentiated by the nine pairs of large vestigial pores in the margin of dorsum; operculum covering the lingula; a cordate vasiform orifice.

Holotype. NIGP205888: a puparium from Hukawng Valley, Kachin State, Myanmar; lowermost Cenomanian, Upper Cretaceous; deposited in Nanjing Institute of Geology and Palaeontology, Chinese Academy of Sciences, Nanjing, China.

Description. A whitefly puparium is enclosed in a transparent, yellow amber piece approximately 13.0 mm in length and 9.5 mm in width. Two flies, an elytron, a thrip, and unidentified insect larvae are preserved as synbioinclusions.

Puparium specimen pale. Body broadly oval, 706 µm long and 452 µm wide (Fig. 3; Figs. 4A–4B). Wax filaments (Fig. 3A, wf) surrounding the whole right margin of the body and 2/3 of the left margin; their texture resembles threads, jointed, forming fringed plates, partially preserved, with length varying from 75 to 389 µm; each plate rises from both vestigial pores and its intermedial areas. *Dorsum* –submarginal area with nine pairs of vestigial pores (Fig. 3, vp; Figs. 4C–4D, vp) plus one in the anterior margin of the body, each 22–38 µm at the widest section. Papillae (Fig. 3, pa; Figs. 4C–4D, pa) are rounded, 3–7 µm, aligned as a row along the submargin, surrounding the internal border of the vestigial pores. Cephalothoracic suture (Fig. 3, cts) is a flat horizontal line immediately above the mouthpart level. Transversal moulting sutures (Fig. 3, tms) visible, not reaching the submarginal area. Longitudinal moulting suture absent. Longitudinal subdorsal furrow (Fig. 3, lsf) present, connecting cephalothoracic suture and transversal suture. Abdominal segments with visible sutures separating them (Fig. 3 as roman numerals; Fig. 4B, as), lengths of segments along midline as follows: I 28 µm, II and III 34 µm, IV 46 µm, V 32 µm, VI 39 µm, VII 33 µm, VIII 14 µm. Vasiform orifice (Fig. 3, vo; Fig. 4E, vo) sclerotised, cordate, located 25 µm from the posterior margin, transversal diameter 50 µm, longitudinal length 42 µm. Operculum (Fig. 3, op; Fig. 4E, op) sclerotised, cordate, transversal diameter 30 µm and 19 µm long. Lingula not visible, probably contained within the operculum. Caudal furrow beardly visible, 25 µm long. *Venter*–antennae (Fig. 3, an; Fig. 4F, an) 40 µm long, with two segments: basal of 13 µm long and apical 30 µm long. Clypeolabral shield (Fig. 3, cl; Fig. 4F, cl) 89 µm long and 72 µm wide, with anterior and posterior arms visible. Labium is not clearly defined. Vestigial legs with two segments, coxa differentiated, lengths for right and left respectively as follows: foreleg (Fig. 3, fl; Fig. 4F, fl) 45 and 41 µm, right mid leg (Fig. 3, ml; Fig. 4F, ml) 58 µm and posterior leg (Fig. 3, pl; Fig. 4B, pl) 61 and 58 µm. *Setae arrangement–* not observed.

Remarks. The species is assigned to Aleyrodinae based on the following characters: (i) absence of claw, (ii) no trace of compound pores, and (iii) no trace of lingula projecting posteriorly from vasiform orifice (Manzari & Quicke, 2006). Additionally, in this case, *C. mranmaense* presents vestigial pores along the margin, accompanied with papillae, which are more common for Aleurodicinae species (*i.e., Aleuroclava*).

The primary distinguishing feature of *C. mranmaense* sp. nov. is the presence of clear areas along the submargin of the dorsum with a row of cuticular protrusions. Two possible structures that fit both the position and shape are papillae as the cuticular protrusions, and simple pores as the ‘clear’ areas. Based on this hypothesis, *C*. *mranmaense* combines two morphological characters that are uncommon in extant species. Regarding the presence of simple pores, it can be compared to species of *Dialeuropora* Quaintance et Baker, 1917, which possess large simple pores along the submargin of the dorsum (Martin, 1999). However, a key difference is the number of pairs of pores: *Dialeuropora* species typically have five pairs, whereas *C*. *mranmaense* displays nine. The geographic distribution of *Dialeuropora* species is also notable, with occurrences in tropical regions of Africa, the Palearctic zone of eastern Asia, and primarily in the Oriental region, including Myanmar (Martin, 1999; Martin & Mound, 2007; Dubey & David, 2012).

The papillae are commonly found in genera such as *Trialeurodes* Cockerell, 1902 and *Bemisia* Quaintance & Baker, 1914, among others (Mound & Halsey, 1978). The particular knoblike shape of papillae can be found in *Trialeurodes merlini* (Bemis, 1904), although the distribution is not restricted to the dorsal submargin and does not form a single row. Species such as *Trialeurodes madroni* (Bemis, 1904) and *T. bemisae* Russell, 1948 present a single row of papillae associated with disc pores, but in both cases, the disc pores are smaller than the papillae. Another significant similarity with those species is the reduced number of sutures.

**Table:** 

Subfamily Aleurodicinae Quaintance & Baker, 1913

**Table:** 

Genus: *Cenomaleurodicus* gen. nov.

Type species. *Cenomaleurodicus multiporus* sp. nov.; here designated

LSID: urn:lsid:zoobank.org:act:74069382-0EC1-45A6-9B1B-119FAA77CB12

Etymology. The generic name is a combination of Cenomanian, referring to the geological age within the Late Cretaceous period, and the generic name *Aleurodicus* Douglas, 1892. Gender: masculine.

Diagnosis. Margin dentate; glandular setae present in submargin band extending beyond the margin; longitudinal moulting suture extending from anterior body margin to posterior margin of metathorax; lingula not completely covered by the operculum (posterior section visible).

**Table:** 

**Figure 5:**
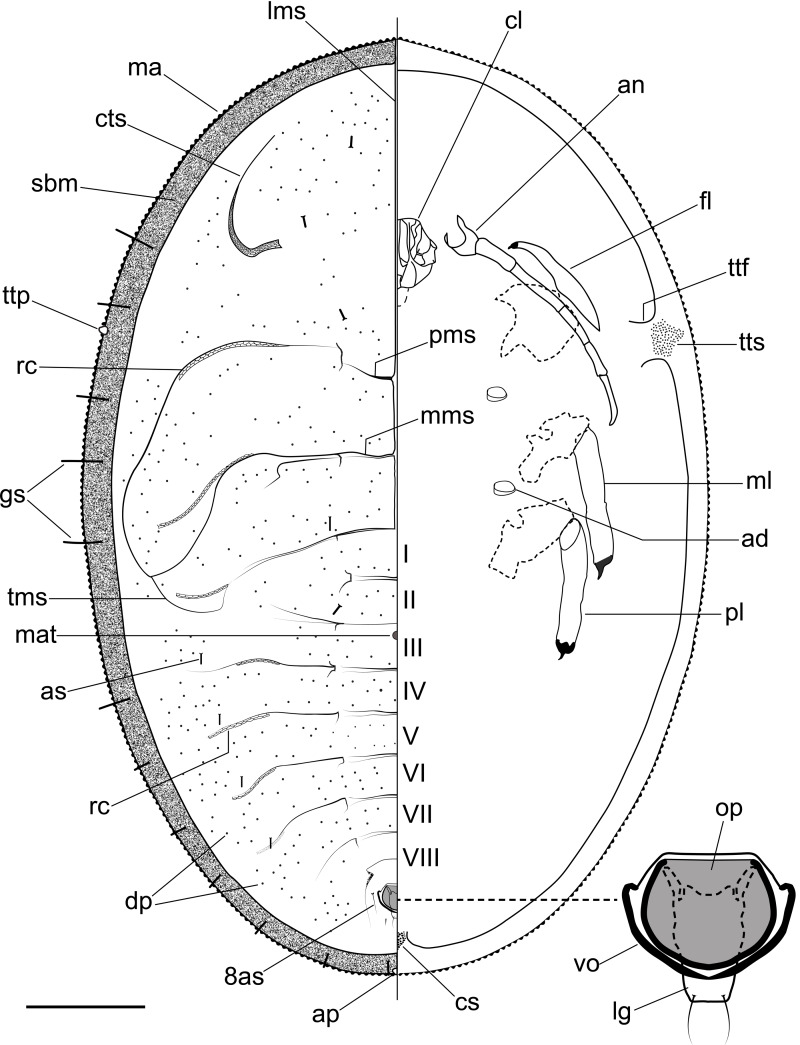
Reconstruction drawing of *Cenomaleurodicus multiporus* gen. et sp. nov., a puparium mspecimen with dorsal view in left side and ventral view in right side. Abbreviations in alphabetical order: (an) antenna, (ap) anal pore, (as) adhesive sac, (as) seta of abdominal segment, (cl) clypeolabral shield, (cs) caudal stipples, (ctsI) anterior lobe of cephalothoracic suture, (ctsII) posterior lobe of cephalothoracic moulting suture, (dp) dorsal pore, (fl) fore leg, (pl) posterior leg, (lg) lingula, (lms) longitudinal moulting suture, (ma) margin, (mat) mesial abdominal tubercule, (ml) mid vestigial leg, (mms) meso+metathorax suture, (op) operculum, (pms) pro+mesothorax suture, (pl) posterior leg, (rc) rachis , (sbm) submarginal band, (sms) submarginal band seta, (tms) trasversal moulting suture, (ttf) thoracic tracheal fold, (ttp) thoracic tracheal pore, (tts) thoracic tracheal stipples, (vo) vasiform orifice, (8as) seta of abdominal segment VIII. Scale bar = 200 µm.

**Figure 6:**
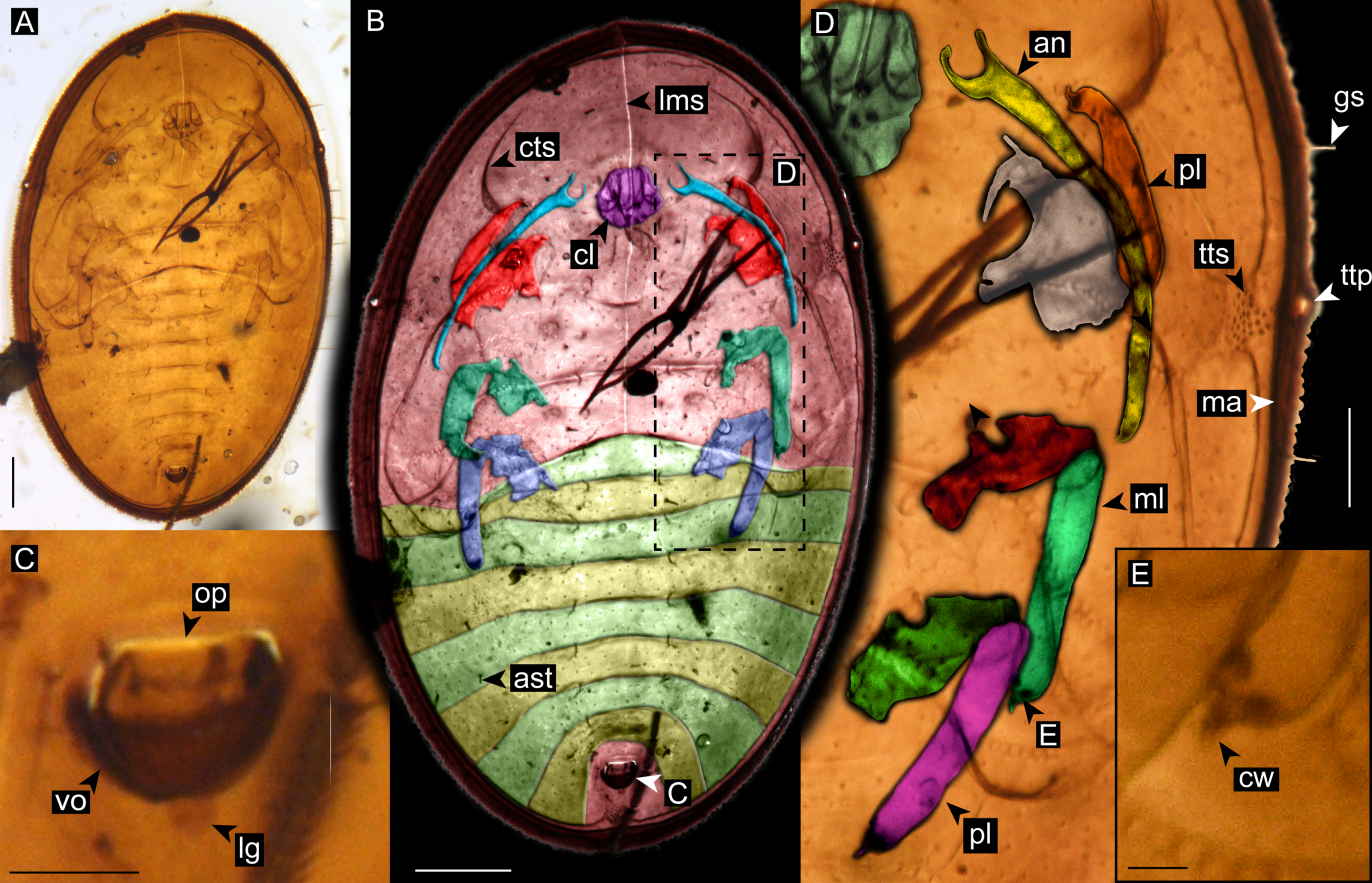
Microphotographs of *Cenomaleurodicus multiporu* gen. et sp. nov. puparium specimen with amplified images. (A) Body, (B) section of thorax and anterior abdominal segments, (C) section of submargin and margin of thorax, (D) abdominal segment VIII with anal apparatus. Abbreviations in alphabetical order: (an) antenna, (ap) anal pore, (as) adhesive sac, (ast) abdominal segment setae, (cl) clypeolabral shield, (cs) caudal stipples, (cf) caudal forrow, (ctsI) anterior lobe of cephalothoracic suture, (ctsII) posterior lobe of cephalothoracic moulting suture, (lg) lingula, (lms) longitudinal moulting suture, (mat) mesial abdominal tubercule, (mms) meso+metathorax suture, (op) operculum, (pms) pro+mesothorax suture, (rc) rachis, (sms) submarginal band seta, (tms) transversal moulting suture, (ttf) thoracic tracheal fold, (ttp) thoracic tracheal pore, (tts) thoracic tracheal stipples, (vo) vasiform orifice, (2as) seta of abdominal segment II, (8as) seta of abdominal segment VIII. Scale bars: A–B = 200 µm; C = 50 µm; *D* = 100 µm; E = 20 µm.

*Cenomaleurodicus multiporus* sp. nov.
Figs. 5 & 6

LSID: urn:lsid:zoobank.org:act:9E142E74-672D-4804-9984-DB8D633FC889

Etymology. The specific name multiporus is a combination of the Latin noun ‘*multus*’, meaning ‘much’ or ‘many’, and the Ancient Greek ‘*π*ó*ρ*o*ς*’ (póros), meaning ‘pore’ or ‘passage in the body’, due to the large number of pores present over the dorsal disc.

Diagnosis. This species is distinguishable by its lobe-shaped cephalotoracic suture, the large number of pores present on the dorsal disc (>200 pores), and its long antennae.

Holotype. NIGP205889: Puparium from Hukawng Valley, Kachin State, Myanmar; lowermost Cenomanian, Upper Cretaceous; deposited in Nanjing Institute of Geology and Palaeontology, Chinese Academy of Sciences, Nanjing, China.

Description. A whitefly puparium is enclosed in a transparent piece of yellow amber approximately 27.5 mm in length and 15.0 mm in width, along with preserved plant trichomes as synbioinclusions.

Puparium specimen pale, without evidence of wax secretion (Figs. 5; 6A–6B). Body broadly oval, 1,756 µm long and 1,119 µm wide. *Dorsum*–margin (Fig. 5, ma; Fig. 6, ma) with crenulate border, with 7–9 teeth per 100 µm. Submarginal band (Fig. 5, sbm) sclerotised, 49–60 µm wide, with remains of at least 13 pairs of glandular setae (Fig. 5, gs; Fig. 6D, gs), 30–76 µm long, the shortest ones, 30–35 µm long, on the posterior abdominal segment. The thoracic tracheal pore (Fig. 5, ttp; Fig. 6D, ttp) is evident on the margin of the prothorax. Longitudinal moulting suture (Fig. 5, lms; 6B, lms) on the dorsum of the head extending from the anterior margin of the head to the posterior margin of the metathorax. Cephalothoracic suture (Fig. 5 cts; Fig. 6B, cts) is not jointed to the longitudinal moulting suture in the anterior section, lateral-posteriorly curved on each side shaped as lobes, without reaching the submargin, with rachis (Fig. 5, rc) on the curve. Transverse moulting suture (Fig. 5, tms) curved shape, not reaching the submarginal line, with two additional sutures connected in the internal area: the anterior one is delimiting the pro–and the mesothorax (Fig. 5, pms), and the posterior suture delimits the meso–and the metathorax (Fig. 5, mms), with vestigial rachis present in curved section. Longitudinal subdorsal furrows absent. Abdominal segments (Fig. 5, roman numerals), with sutures delimitating each one of them, not reaching the submarginal band, with vestigial rachis in the submedian section; lengths of segments along midline as follows: I and II 90 µm each, III 88 µm, IV 85 µm, V 81 µm, VI 79 µm, VII 71 µm, VIII 111 µm. Vasiform orifice (Fig. 5, vo; Fig. 6C, vo), cordate, with sclerotised margins only on the posterior half, transversal diameter 80 µm, longitudinal diameter 62 µm. Operculum (Fig. 5, vo; Fig. 6C, op) cordate, with anterior margin unsclerotized, 55 µm long and 66 µm wide, covering two-thirds of the lingula. Lingula (Fig. 5, lg; Fig. 6C, lg) 67 µm long and 42 µm wide, with the distal section not covered by the operculum, with two flagellate setae in the apex. Caudal furrow is not clearly differentiated from the caudal tracheal fold, 82 µm long. *Setae arrangement*–Setae of dorsal disc partially preserved, stout with broken apex, resembling glandular seta type. Distribution on head two pairs present in the area delimited by the by the cephalothoracic suture; on thorax one pair anterior to pro+mesothorax suture, one pair anterior to meso+metathorax suture, length 17–25 µm; on abdomen, absent on segment I, one pair in the central area of segment II, one seta on each lateral area near the posterior margin of abdominal segments III (Fig. 5 ast; Fig. 6B, ast) to VII, 19–33 µm long; segment VIII with a flagellate seta positioned on each lateral side, at the base of anterior margin of the vasiform orifice (Fig. 5, 8as). *Pores –* Dorsal disc pores (Fig. 5, dp) are scattered along the area, >200 pores, each 4–5 µm. Medial abdominal tubercle (Fig. 5, mat) presents on abdominal segment III. *Venter*–antennae (Fig. 5, an; Fig. 6D, an) with at least six well-defined antennomeres, total length 454–517 µm, segment I 66 µm long, II 94 µm long, III 75 µm long, IV 140 µm long, V 50 µm long, VI 92 µm long. Clypeolabral shield (Fig. 5, cl; Fig. 6B, cl) 127 µm long and 143 µm wide. Labium is not clearly defined. Vestigial legs composed of a coxa partially developed, plus fusion of trochanter-femur-tibia-tarsus, plus apical sclerotised process that seems to be claws (Fig. 6E, cw); foreleg (Fig. 5, fl; Fig. 6D, fl) 205 and 240 µm long, mid leg (Fig. 5, ml; Fig. 6D, ml) 274 µm long and posterior leg (Fig. 5, pl; Fig. 6D, pl) 259 and 274 µm long. Adhesive sacs (Fig. 5, ad) partially visible on mesothorax and metathorax, one pair in each area. Thoracic tracheal fold (Fig. 5, ttf) short, extended from margin 66–75 µm, with a cluster of stipples (Fig. 5, tts). Caudal tracheal fold with a similar length of caudal furrow, with around 18 stipples (cs) clustered on the posterior side.

Remarks. The anatomy of this fossil presents an interesting mosaic of features that, to some degree, contradict the traditional morphological delimitation of the extant subfamilies Aleyrodinae and Aleurodicinae, including the absence of compound pores on the dorsum, a large tongue-shaped lingula extending posteriorly beyond the vasiform orifice, and the presence of claws. However, this issue is not a novelty, as we discuss below.

The subfamily Aleyrodinae is defined by the absence of compound pores in the dorsum, the absence of claws, and a lingula usually enclosed in the vasiform orifice area; in contrast, Aleurodicinae can be separated by the usual presence of compound pores in the dorsum, the presence of claws and a lingula that extends beyond the vasiform orifice posteriorly (Martin, 1987; Martin, 1999; Martin, 2005; Evans, 2008). Nevertheless, recently described species and genera from Aleurodicinae, challenge that delimitation, overlapping states of characters that were restricted to Aleyrodinae (Martin & Streito, 2003; Gillespie, 2006; Martin, 2008).

Tarsal claws may be present or absent in Aleurodicinae (*e.g.*, *Stenaleyrodes* Takahashi, 1938 and *Palaealeurodicus* Martin, 2008), whereas no species of Aleyrodinae present such claws (Martin, 1999; Martin, 2008; Gillespie, 2006; Manzari & Quicke, 2006). A similar situation occurs with compound pores: most Aleurodicinae species lack these pores and may or may not present tarsal claw (*e.g.*, *Dialeurodicus* Cockerell, 1902, *Stenaleyrodes* Takahashi, 1938, *Synaleurodicus* Solomon, 1935, and *Pseudosynaleurodicus* Gillespie, 2006) (Martin, 2004; Martin, 2008; Gillespie, 2006). Furthermore, the number of setae on the lingula varies in Aleyrodinae between 0 and 1 pair (Manzari & Quicke, 2006), while in Aleurodicinae it varies from 0 to 2 pairs (Gillespie, 2006).

The single character that appears stable across both subfamilies is the grade of extension of the lingula regarding the vasiform orifice. The lingula is never projected beyond the posterior margin of the vasiform orifice in Aleyrodinae, and always extends beyond the posterior margin in Aleurodicinae (Gillespie, 2006; Manzari & Quicke, 2006). This is the case for *C. multiporus*, which, in conjunction with the presence of claws and the number of lingular setae, allows this taxon to be assigned to the subfamily Aleurodicinae, representing the first member of this subfamily described from the puparial stage.

In this taxonomic context, two species resemble the morphology of *C. multiporus*. *Dialeurodicus caballeroi* Martin, 2004 presents a large number of pores on dorsal disc, length of antenna reaching the middle leg, and bi-segmented legs (Martin, 2004). The main differences with *C. multiporus* cover, first, the type of dorsal pores: in *D. caballeroi* are geminate type (modified 8-shaped pores), which it is not possible to corroborate for *C. multiporus*. Second, the lingula in *D. caballeroi* presents four setae in the tip of lingula and does not extend beyond the vasiform orifice (Martin, 2004). In contrast, in *C*. *multiporus* only two are visible, and the lingula extends beyond the vasiform orifice. *Pseudosynaleurodicus nigrimarginatus* Gillespie, 2006 is similar to *C*. *multiporus* in the following features: bi-segmented legs; lingula projected beyond the posterior margin of vasiform orifice, with 1 pair of long setae on the apical area; sclerotised submarginal area, bearing 15 or 16 pairs of setae (Gillespie, 2006). The main differences are the short antennae, reaching up to the fore coxae, and the lack of pores on dorsum.

## Discussion

For the first time, anatomy of fossilised puparium specimens is available for analysis, with both ventral and dorsal surfaces visible. Taxonomy of extant whiteflies is based mainly on morphological characters of the 4th (last) immature stage, the puparium, as opposed to the characteristics found in adults. This is due to the sessile nature of puparia that makes them easy to collect, and the high diversification of morphological structures for taxonomy, in comparison with the adult and the first three immature stages (Martin, 2003; Hodges & Evans, 2005; Manzari & Quicke, 2006; Gullan & Martin, 2009; Hidayat et al., 2019).

In contrast, the taxonomy of extinct species, particularly those from the Mesozoic, is mainly based on adult specimens (Weigelt, 1940; Rietschel, 1983; Matthews & Telka, 1997; Szwedo et al., 2019; Drohojowska et al., 2023). Despite the existence of several puparia fossils, the formal descriptions and their corresponding taxonomical placements are less abundant than those based on adults. This disparity may be related to behavioural and taphonomic factors. Immature stages 2 to 4 are sessile and remain attached to host plants (Martin, 2004), which may reduce their likelihood of being directly intercepted and trapped in resin, in contrast to mobile adults. In addition, once preserved in compression fossils, many diagnostic characters may be obscured by the surrounding matrix. Furthermore, their small size makes them more easily overlooked during amber collection, contributing to a potential collection bias.

To date, five adult-stage species have been described from Burmese amber: *Aleurodicus burmiticus* Cockerell, 1919; *Burmodicus cretaceous* Chen et al., 2021 and *B. monlyae* Chen & Zhou, 2022 (Aleurodicinae); and *Burmoselis evelynae* Shcherbakov, 2000 and *Paraburmoselis kachinensis* Chen et al., 2020 (Bernaeinae) (Cockerell, 1919; Shcherbakov, 2000; Chen et al., 2020; Chen et al., 2021; Chen et al., 2022). There is no Aleyrodinae species, and therefore, the single taxonomic connection could be between Aleurodicinae species, *i.e., Cenomaleurodicus multiporus* + *A*. *burmiticus* + *B. cretaceous* + *B. monlyae*. While a definitive taxonomic link between those species is not currently feasible, due to morphological differences between developmental stages, the possibility that the immature form of *Cenomaleurodicus multiporus* represents the puparial stage of one of the previously described species cannot be ruled out.

The formal species described from puparia are restricted to three, all preserved through mineralisation. *Aleurochiton petri* is described from a specimen attached to *Acer* sp. (Aceraceae) leaves preserved in the Late Pliocene (Piacenzian) claystone deposits of the Kiesgrube Fr. Bauer, Neu-Isenburg, Hesse, Germany (Rietschel, 1983). The specimen exhibits cephalothoracic, promesothoracic and transverse sutures, abdominal segments I to VIII and remains of the vasiform orifice, but no cuticular structures are visible. The second species, *Praealeurolobus indicus* was described from the Pliocene Rajdanda Formation of Jharkhand, north-eastern India, where the specimen is attached to a fossil leaf of Malvaceae (Drohojowska et al., 2023). In this case, besides the marginal band, transversal and longitudinal moulting sutures, the specimens exhibit mesothoracic and caudal setae. The last species, *Miotetraleurodes novaezelandiae* was described from the Hindon Maar Complex, a Miocene fossil Lagerstätte within the Dunedin Volcanic Group in Otago, South Island, New Zealand (Drohojowska et al., 2024). These specimens preserved the submarginal, transversal and longitudinal moulting sutures, plus conical teeth on the body margin, and a band of grooved striae along the submargin, with no setae visible. These species are described solely on the basis of dorsal morphology, with no information on chaetotaxy or wax gland systems. A comparison between the species described here and the three species mentioned above is constrained by the limitations of the available anatomical data and the considerable age difference separating them. Nonetheless, it is important to mention these taxa to highlight the significance of amber-preserved fossils and the still poorly understood morphology of the puparial stage.

As noted throughout this paper, differentiating the whiteflies subfamilies based on puparium characters is challenging. The key features commonly used to distinguish Aleyrodinae from Aleurodicinae, with the latter indicated in parentheses, include the absence of claws (claws present), a lingula enclosed within the vasiform orifice (lingula extending posteriorly beyond the orifice), and the absence of dorsal compound pores (pores present) (Martin, 1999; Martin, 2004; Martin, 2005). In this context, the fossils described here contribute to the taxonomic discussion of subfamilies. *Cretacerifera mranmaense* and *Palaeoaleuroclava grandirostrata* align with the conventional definition of Aleyrodinae, whereas *C. multiporus* presents a conflicting yet not uncommon combination of traits that complicates the circumscription of Aleurodicinae. It is possible to infer i) that the lack of compound pores and the number of lingulan setae may not be as relevant when the specimen presents tarsal claws, and ii) the extension of lingula regarding the vasiform orifice, although it seems weak, is the best approach to subfamily differentiation. In the case of *C. multiporus,* both the presence of a claw and the projection of the lingula placed it in Aleurodicinae.

The establishment of a new subfamily was considered but ultimately rejected for three main reasons. First, the fossil displays a mosaic of characters shared with both extant subfamilies but lacks unique, autapomorphic features that would clearly define a new subfamily. Second, given the ongoing uncertainty surrounding subfamily delimitations in extant whiteflies, and the lack of a robust phylogenetic framework, erecting a new subfamily based on a single fossil specimen would likely add further instability to the classification. Lastly, direct correspondence between fossil adult whiteflies and puparial stages is currently not possible due to the lack of overlapping diagnostic characters and the absence of specimens preserving both life stages. We therefore prefer to place the fossil within an existing subfamily while highlighting its unusual character combination in the remarks and discussion, which we believe is the most conservative and informative approach.

The modification of pretarsi as claws or pads can be traced back to at least the mid-Cretaceous period, and both characters were found simultaneously, within the same geological period. It is likely to assume that the modification of the pretarsi occurred in Early Cretaceous or earlier. Moreover, the finding of a not-so-common mosaic of characters, as the presence of claws and the extension of the lingula with the lack of compound pores, exhibited by *C. multiporus* (Aleurodicinae), could mean that the presence or absence of compound pores could be a derived character, and it did not evolve at the same time as the modification of the pretarsi. Another relevant finding is the presence of wax strands and the cuticular structures that produce them, which could be considered the primary stages in the evolution of specialised glandular structures that are currently highly complex. The development of cuticular glands could also be the result of the response to changes in host plant biochemistry and physiology, driven by modifications in phloem sap composition (Sandstrom & Moran, 1999; Douglas, 2006; Broussard et al., 2023). The wax production aspect is also relevant to understanding the interaction between the insect and its environment, which is typically influenced by abiotic factors (*e.g.*, temperature, humidity) and biotic factors (*e.g.*, natural predators, parasites and parasitoids) (Byrne & Hadley, 1988; Labandeira & Li, 2021). Although the evolutionary drivers or mechanisms underlying wax structures remain unclear, these features are relevant for taxonomic purposes, including their use in genus level demilitations (Manzari & Quicke, 2006). Taken together, the morphological mosaic observed in *C. multiporus* and the inferred evolutionary patterns of cuticular glands underscore the complexity of whitefly evolution and the need for further integrative studies.

Amber-inclusion specimens are usually well preserved, but the degree of amber opacity poses a challenge for morphological studies. Previous studies on insect fossils of Coleoptera, Diptera, Hymenoptera and other groups, using the Synchrotron X-ray computed microtomography–SRµCT technique, have provided details in micron-sized structures of exoskeleton and internal anatomy, with implications for the taxonomic hypotheses (*e.g.*, Soriano et al., 2010; van de Kamp, 2014). The equipment and techniques employed in the present study did not allow us to obtain detailed information on structures taxonomically relevant, such as vestigial pores, tubercles, papillae, or the chaetotaxy of the antennae and legs. We estimate that substantial morphological information remains to be analysed, information that SRµCT technology may be able to recover. Given its potential to resolve fine anatomical details, future application of SRµCT to amber-preserved whiteflies could significantly enhance taxonomic and evolutionary interpretations.

## Conclusions

Unlike other arthropod groups, information on immature Aleyrodidae fossils is scattered and morphologically restricted. For the first time, the morphology of fossil puparia is analysed and discussed at a level comparable to that of extant specimens. The taxa described above represent the oldest and well-preserved-body fossils of Aleyrodidae puparia to date, providing information about the morphology of the group. Amber preservation allows exceptional access to both ventral and dorsal structures, enabling comparisons with extant puparia and refining the morphological definition of immature stages.

There is an evident disadvantage to detecting cuticular characters in fossils, which affects the reliability of the assigned subfamilies for the proposed species. Nevertheless, the possibility of engaging with subfamily assignment is now open, thanks to the first availability of amber-preserved fossils. These specimens hold substantial informational potential, and the application of advanced imaging techniques may allow a reassessment of current subfamily classifications.

The specimens described here represent a significant step forward in understanding the evolution, diversity, and morphology of whitefly puparia. Therefore, the present study describes the first three whitefly species based on puparium specimens from the Cretaceous, thereby filling a gap in the fossil record of Aleyrodidae.

## Additional Information and Declarations

### Data Availability

The following information was supplied regarding data availability: This manuscript does not include any supplementary data. All high-resolution images of the fossil specimens are provided directly within the main text to ensure full accessibility and transparency of the material.

### New Species Registration

The following information was supplied regarding the registration of a newly described species: Genus name: urn:lsid:zoobank.org:act:50847E47-A3B7-4D91-9989-5B366ED4DA47 Species name: urn:lsid:zoobank.org:act:46856C02-90A4-453A-9E49-E19B8B7AB8AB Genus name: urn:lsid:zoobank.org:act:BA3A9BAD-75DC-4403-B185-84987D2B5C88 Species name: urn:lsid:zoobank.org:act:E1FBA0DF-041C-4D0C-856D-9F530325B9C7 Genus name: urn:lsid:zoobank.org:act:74069382-0EC1-45A6-9B1B-119FAA77CB12 Species name: urn:lsid:zoobank.org:act:9E142E74-672D-4804-9984-DB8D633FC889 Publication LSID: urn:lsid:zoobank.org:pub:pub:8E9CE1A7-2833-440F-B9E9-BBCE5B83ABC4.
